# PREOPERATIVE COMPUTED TOMOGRAPHY ANGIOGRAPHY IN MULTIDISCIPLINARY PERSONALIZED ASSESSMENT OF PATIENT WITH RIGHT-SIDED COLON CANCER: SURGEON AND RADIOLOGIST POINT OF VIEW

**DOI:** 10.1590/0102-672020220002e1679

**Published:** 2022-08-26

**Authors:** Stepan GRYTSENKO, Ihor DZYUBANOVSKY, Ivanna HRYTSENKO, Anatoliy BEDENIUK

**Affiliations:** 1 I. Y. Horbachevsky National Medical University, Department of Surgery Nº 1 with Urology and Minimal Invasive Surgery by L.Ya. Kovalchuk – Ternopil, Ukraine;; 2 Medical Center “Omega”, Radiological Department – Kyiv, Ukraine.

**Keywords:** Colonic Neoplasms, Anastomotic Leak, Tomography, X-Ray Computed, Angiography, Neoplasias do Colo, Fístula Anastomótica, Tomografia Computadorizada por Rx, Angiografia

## Abstract

**BACKGROUND:**

3D-CT angiography has made it possible to reach a qualitatively new level in the determination of treatment tactics for patients with colorectal cancer.

**AIMS:**

This study aimed to analyze the clinical and radiological aspects that need to be discussed before surgery by a multidisciplinary team in patients with right-sided colon cancer.

**METHODS:**

This study involved 103 patients with colorectal cancer who underwent preoperative 3D-CT angiography from 2016 to 2021

**RESULTS:**

All patients underwent radical D3 right hemicolectomy. The median quantity of removal lymph nodes were 24.71±10.04. Anastomotic leakage was diagnosed in one patient. We have identified eight most common types of superior mesenteric artery. The ileocolic artery crossed the superior mesenteric vein on the anterior surface in 64 (62.1%) patients and on the posterior surface in 39 (37.9%). In 58 (56.3%) patients, the right colic artery was either absent or was a nonindependent branch of superior mesenteric artery. The distance from the root of the superior mesenteric artery to the root of the middle colic artery was 37.8±12.8 mm and that from the root of the middle colic artery to the root of the ileocolic artery was 29.5±15.7 mm. The trunk of Henle was above the root of the middle colic artery in 66 (64.1%) patients, at the same level with the middle colic artery in 16 (15.5%), and below the middle colic artery in 18 (17.5%) patients.

**CONCLUSIONS:**

Preoperative analysis of 3D-CT angiography is a key pattern in assessment of vascular anatomy and can potentially show the complexity of future lymphadenectomy and reduce the risk of anastomotic leakage.

## RESUMO

**RACIONAL:**

A angiografia 3D-TC permitiu alcançar um nível qualitativamente novo na determinação de táticas de tratamento para pacientes com câncer colorretal.

**OBJETIVO:**

Analisar os aspectos clínicos e radiológicos que precisam ser discutidos antes da cirurgia por uma equipe multidisciplinar em pacientes com câncer de cólon direito.

**MÉTODOS:**

Analisar 103 pacientes com câncer colorretal submetidos à angiotomografia 3D pré-operatória entre 2016 e 2021.

**RESULTADOS:**

Todos os pacientes foram submetidos à hemicolectomia direita radical D3. A quantidade mediana de linfonodos removidos foi de 24,71±10,04. Deiscência de anastomose foi diagnosticada em 1 paciente. Identificamos 8 tipos mais comuns de artéria mesentérica superior. Em 64 pacientes (62,1%) a artéria ileocólica cruzou a veia mesentérica superior na face anterior e em 39 (37,9%), na face posterior. Em 58 pacientes (56,3%) a artéria cólica direita estava ausente ou era um ramo não independente da artéria mesentérica superior. A distância da raiz da artéria mesentérica superior à raiz da artéria cólica média foi de 37,8±12,8 mm e a distância da raiz da artéria cólica média até a raiz da artéria ileocólica foi de 29,5±15,7 mm. Em 66 pacientes (64,1%) o tronco de Henle estava acima da raiz da artéria cólica média, em 16 (15,5%) o tronco de Henle estava no mesmo nível da artéria cólica média e em 18 pacientes (17,5%) o tronco de Henle estava abaixo da artéria cólica média.

**CONCLUSÕES:**

A angiografia 3D-CT pré-operatória é um padrão chave na avaliação da anatomia vascular e pode potencialmente mostrar a complexidade de uma futura linfadenectomia e reduzir o risco de deiscência da anastomose.

## INTRODUCTION

The incidence of anastomotic leak (AL) after right hemicolectomy is relatively low, in comparison with left-sided/rectal colorectal cancer (CRC). In 2015, the European Society of Coloproctology (ESCP) audited right colectomy and ileocecal resection, collecting prospective data on 3,208 patients across 284 centers in 39 countries. The overall AL rate was 8.1%^11^. This is due to a more stable blood supply. However, different anatomical variations can have a significant impact on the duration of surgery and cause the technical complexity of its implementation. The need for standardization is still debated in the literature of Eastern D3 lymphadenectomy and Western embryologically oriented complete mesocolic excision with central vascular ligation (CME/CVL)^5,8^.

In contrast to left and rectal cancer surgery, where the inferior mesenteric artery is the most important reference point, there are several such points, including the superior mesenteric vein (SMV), truncus Henle (TH), and the branches of the superior mesenteric artery (SMA). Not uncommon anatomical variability of the abovementioned vessels causes a higher percentage of conversions to open operations and increases intraoperative time and intraoperative blood loss^9,12^. It is difficult, in the scientific world of the 21st century, if not impossible, to say anything new in surgical anatomy of the abdominal cavity. However, with widely used in clinical practice, contrast-enhanced computed tomography (CT) has made it possible to reach a qualitatively new level in preoperative diagnosis and determination of treatment tactics for patients with CRC. Routine use of CT angiography allows a detailed analysis of each clinical case in the preoperative stage and identifies various anatomical nuances that may affect the operation^6,7^.

The aim of this article was to analyze the clinical and radiological aspects that usually need to be discussed before surgery by a multidisciplinary team in patients with right-sided colon cancer.

## METHODS

This study was carried out a comparative analysis of 3D-CT angiography data with intraoperative data. A detailed analysis of the anatomy of the branches of the SMA and its relationship with the surrounding structures was done in order to explore the nuances that may complicate and increase the time during right hemicolectomy with CME/CVL. The relationship between the anatomical features of the structure of SMA and postoperative complications was also investigated.

### Description of patients

We included 103 patients (56 males and 47 females; mean age 64.2±11.6) with CRC who underwent preoperative 3D-CT angiography at Ternopil University Hospital from 2016 to 2021. The exclusion criteria were stage IV process and locally advanced forms of cancer. The informed consents were obtained from all patients. This study was passed by the Ethics Commission of Ternopil National Medical University (no. 43).

### Measurements

In this study, the following objectives were set: determine the type of SMA; determine the distance from the root of the SMA to the root of the middle colic artery (MCA), the distance from the root of the MCA to the root of the ileocolic artery (ICA); variant structure of the right colic artery (RCA); and the relationship between MCA and gastrocolic TH; and explore different variants of TH confluence.

The distance between the vascular structures was measured in the frontal plane using a linear measurement. Anatomical features of the structure of SMA branches were determined in the arterial phase and venous structures of TH in the venous phase and compared the ratio of MCA and TH using Fusion.

### Scan protocol

3D-CT angiography was performed using a Philips Brilliance 64 CT machine with IV contrast (100 mL of iodinated contrast agent [370 mg/mL]). Contrast was injected into the ulnar vein at a rate of 4.5 mL/s. The bolus tracking method was used for scanning. Arterial phase scanning automatically began when the contrast in the abdominal aorta at the level of the abdominal trunk reached 180 HU. The 64-slice multidetector CT scanner (MDCT) can generate 0.75-mm slices that can be reconstructed into a 0.5-mm image. Therefore, in order to obtain high-quality CT angiography for preoperative analysis, a scanning protocol should be maintained: sublingual nitrate intake, high contrast rate (4–5 mL/s), early arterial phase (20–30 ‘), stress reduction (80–100 kV), and doubling the mAs. Image processing was performed using 3D volume rendering technique (VRT).

### Statistical analysis

Ordinal data were calculated using the median. All calculations were performed using the Statistica version 64 software.

## RESULTS

All patients underwent local radical right hemicolectomy with CME/CVL and R0 resection.

The median quantity of removal lymph nodes was 24.71±10.04 (range 13–58). Positive lymph nodes were revealed in 38.7% of cases. The incidence of metastatic lymph nodes was 38.7% in D1 zone, 3.2% in D2 zone, and 9.7% in D3 zone. Mean operative time was 82 min (range 63–130). Median intraoperative blood loss was 70 mL (range 32–280). No patients required intraoperative blood transfusion. Postoperative complications were developed in seven patients. AL was diagnosed in one patient on postoperative day 8 for whom relaparotomy, lavage, and end stoma were performed (Figure 5). Unfortunately, on the first day after patient discharge from the hospital, he died from massive thromboembolic complication, despite maintaining prophylaxis therapy. One patient suffered from paralytic ileus in an early postoperative period. Median staying in hospital after operation was 8.4 days.

The SMA was present in 100% of cases. Compared with the widely used Zebrowski classification of the inferior mesenteric artery, we could not find a common classification of anatomical variations of SMA. We have identified eight types that are most common in practice:

- **Type A** – MCA, RCA, and ICA deviate classically independently of each other from the main SMA trunk.
- **Type B** – RCA is absent.
- **Type C** – RCA deviates from ICA.
- **Type D** – RCA deviates from the right branch of the MCA or the main trunk of the MCA.
- **Type E** – Classical type A + the presence of additional MCA (AMCA)
- **Type F** – Right and left MCA branches deviate separately from the main SMA trunk.
- **Type G** – MCA and ICA have a common trunk and RCA is absent.
- **Type H** – RCA deviates from ICA + AMCA.

**Figure 1:**
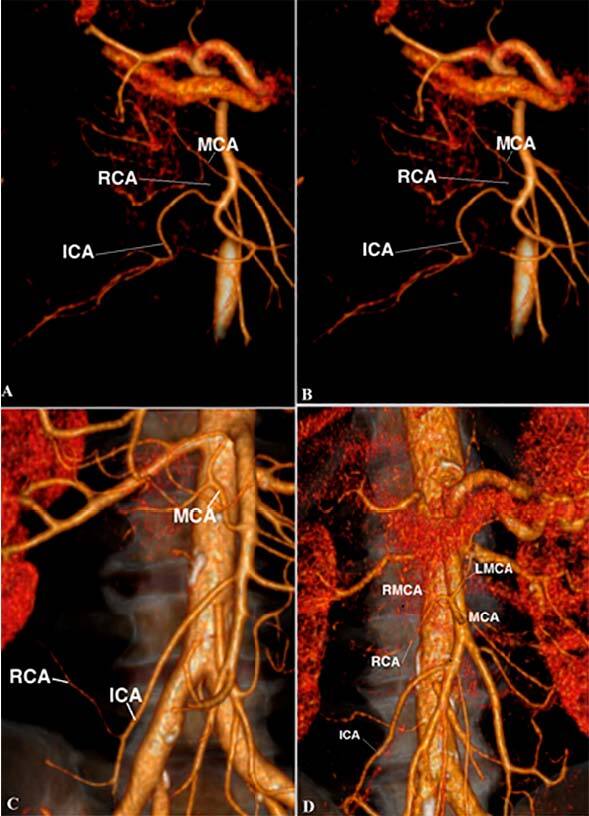
Types A, B, C, and D of superior mesenteric artery.

MCA: middle colic artery; RCA: right colic artery; ICA: ileocolic artery.

**Figure 2:**
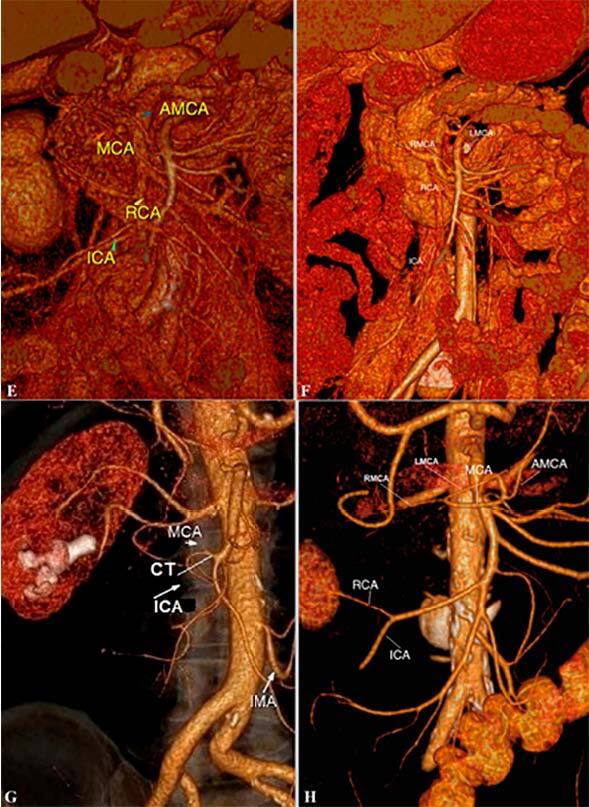
Types E, F, G, and H of superior mesenteric artery.

MCA: middle colic artery; RMCA: right middle colic artery; LMCA: left middle colic artery; AMCA: additional middle colic artery; RCA: right colic artery; ICA: ileocolic artery; CT: common trunk.

Our analysis showed that type A was detected in 27 (25.9%) patients, type B in 22 (21.4%), type C in 20 (19.2%), type D in 12 (11.6%), type E in 9 (8.7%), type F in 9 (8.7%), type G in 3 (2.9%), and type H in 1 (0.9%) patient (Figures 1 and 2). The analysis also showed that in 12 (11.6%) patients, the right hepatic artery deviates from SMA.

The ICA was present in 103 (100%) cases. The ICA crossed the SMV on the anterior surface in 64 (62.1%) cases and on the posterior surface of the SMV in 39 (37.9%) cases.

The RCA is one of the most volatile arterial structures of the SMA system (literature data indicate that it is present in 11–40% of cases)^1,4,12^. According to our selected types of SMA, RCA was absent in 25 (24.3%) and in 33 (32%) patients and deviated from ICA and MCA/RMCA. Accordingly, in 58 (56.3%) patients, RCA was either absent or was a nonindependent branch of SMA.

The MCA was present and originated directly from SMA in 103 (100%) cases. AMCA was present in 10 (9.7%) cases.

The distance from the root of the SMA to the root of the MCA was 37.8±12.8 mm (range 13–65).

The distance from the root of the MCA to the root of the ICA was 29.5±15.7 mm (range 0–80).

**Figure 3:**
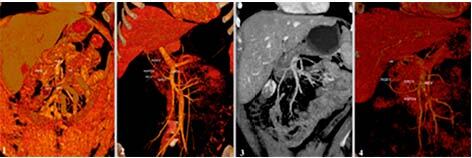
Types of truncus Henle.

MCV: middle colic vein; RCV: right colic vein; AMCV: additional middle colic vein; RGEV: right gastroepiploic vein; ASPDV: anterior superior pancreaticoduodenal vein; ICV: ileocolic vein.

Gastrocolic TH was present in 100 (97.1%) cases and located on the lower edge of the mesentery of the transverse colon, along the head of the pancreas, and flows into the right lateral part of the SMV wall. Our analysis showed that the caliber of TH varied from 3 to 10 mm and its length was 11.5±4.8 mm (range 2–33). Usually, the confluence of TH formed: middle colic vein (MCV), right colic vein (RCV), additional middle colic vein (AMCV), right gastroepiploic vein (RGEV), and anterior superior pancreaticoduodenal vein (ASPDV). Also, we observed a very interesting case where one of the veins which create confluence of TH was ileocolic vein (ICV) (Figure 3). Our analysis of 3D-CT angiograms showed the following type combinations of TH confluence:

- MCV+RCV
- RCV+RGEV+ASPDV
- ICV+RCV+RGEV
- Absence of TH
- MCV+RGEV
- MCV+RGEV+ASPDV+AMCV

Respectively, type 1 was observed in 17 (16.5%) patients, type 2 in 55 (53.4%), type 3 in 1 (1%), type 4 in 3 (2.9%), type 5 in 15 (14.6%), and type 6 in 12 (11.6%) patients (Figure 3). Unfortunately, it was impossible in some cases to create 3D-CT reconstruction of some types of TH due to lack of contrast, incorrect scanning, and various technical features.

**Figure 4:**
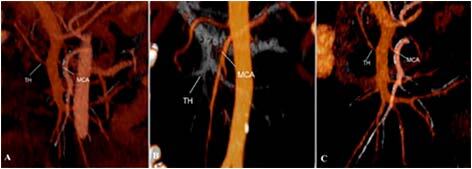
Correlation between root of truncus Henle and middle colic artery. (A) truncus Henle above the root of the middle colic artery; (B) truncus Henle below of the root of the middle colic artery; (C) truncus Henle at the same level with the root of the middle colic artery.

TH: truncus Henle; MCA: middle colic artery.

An important point of preoperative planning is understanding the relationship between TH and MCA. In 66 (64.1%) patients, TH was located above the root of the MCA, in which case the distance between them was 12.38±5.41 mm (range 3–29). In 18 (17.5%) patients, TH was located below the root of the MCA, in which case the distance between them was 10.95±7.1 mm. In 16 (15.5%) patients, TH was located at the same level with the root of the MCA (Figure 4).

## DISCUSSION

**Figure 5:**
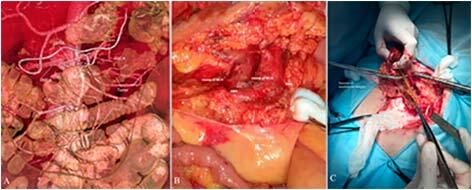
Clinical case of anastomotic leakage: (A) intraoperative photo after D3 lymphadenectomy; (B) retrospective 3D reconstruction; (C) relaparotomy, anastomotic leakage (necrosis of anastomosis margins).

SMA: superior mesenteric artery; SMV: superior mesenteric vein, stump of middle colic artery and middle colic vein.

The well-known concept of right hemicolectomy with CME/CVL, in the past decade, has supplanted the traditional old notion of colon cancer surgery and has improved patients’ 5-year survival^1,5,8^. AL is the most devastating complication in colorectal surgery. Patients who developed an AL had a higher mortality than those who did not, a longer median hospital stay, and a higher 30-day reoperation and 30-day readmission rate^11^. In our study, we observed AL in one patient, which resulted in 30-day mortality (Figure 5). Retrospective analysis of this case showed our mistake. According to the oncology canons, the operation was performed correctly (CME/CVL), but we did not perform an analysis of vascular anatomy before surgery, resulting in irreversible ischemic changes in the anastomotic area and the actual AL.

In the left-sided CRC, the inferior mesenteric artery is the most important landmark to perform D3 lymphadenectomy, while in the right-sided colon cancer surgery, there are several such “central” landmarks: SMV, SMA, ICA, MCA, and TH.

Each right hemicolectomy with CME/CVL started from dissection of SMV and identification of ICA and ICV. Here we do not have any problem. However, interesting is the effect of the course of ICA in relation to SMV on disease-free survival with correspondingly better results in the group of patients where ICA is ahead of SMV^6^. Therefore, a potential group of patients with an ICA course, behind the SMV, requires more precision lymphadenectomy of the apical area. In our study, we found that ICA crossed the SMV on the anterior surface in 64 (62.1%) cases and on the posterior surface of the SMV in 39 (37.9%) cases.

RCA is one of the most volatile arterial structures of the SMA system. Literature data indicate that it is present in 11–40% of cases^1,4,12^. In our study, we found that the weighted mean incidence of RCA was 43.7% from the SMA, 20.4% from the ICA, and 11.6% from the root of the MCA or rMCA, and RCA was absent in 25 (24.3%) cases. Usually, we do not encounter any problems with RCA when implementing the Western concept of CME/CVL and do not pay much attention to it if it is not an independent branch. However, the abovementioned anatomical variants of RCA should be considered when performing the Eastern concept of D3 lymph node dissection (segmental resections — 10 cm from the edge of the tumor)^10^.

The next one key point for performing right hemicolectomy with CME/CVL is TH, especially due to being a special area of the apical lymph nodes. TH is a thin-walled venous trunk that has many different combinations of formation. Traditional TH branches are MCV, RGEV, ASPDV, and aMCV^1,2^. Very often, it is in the TH area due to excessive traction of the mesentery during the allocation of MCA surgeons get bleeding. It is critical to understand the relationship between TH and MCA to prevent damage of this trunk (Figure 4). In our study, we found that TH was located above the root of the MCA (12.38±5.41 mm) in 66 (64.1%) patients, at the same level with the root of the MCA in 16 (15.5%) patients, and was located below the root of the MCA (10.95±7.1 mm) in 18 (17.5%) patients. In the situation if the root of the MCA is above TH, it is safer to start mobilization from the cranial part of the root of transverse colon mesentery, and in cases where the root of the MCA is below TH, then the dissection of MCA should begin from the caudal part of transverse colon mesentery^7^.

CT is a modality of choice for staging of colon cancer and distant metastasis. Magnetic resonance angiography is an expensive method to perform it routinely and preoperatively for every patient. Therefore, CT is optimal for staging and evaluating mesenteric vasculature^8^. However, 3D-CT angiography has several limitations. First, the preoperative CT protocol for patients with colon cancer usually does not include the early arterial phase and, therefore, results in some difficulty in performing adequate 3D reconstruction. Second, the caliber of SMA branches is usually small in diameter and they are not always well visualized on 3D-CT angiograms. In the abovementioned cases, the use of CT in the preoperative analysis of anatomical variants of the structure of SMA branches cannot be performed in 3D mode and should be performed in normal 2D mode^1^.

New era of development personalized strategy could be achieved by virtual reality exploration and planning for precision colorectal surgery, which can provide an enhanced understanding of crucial anatomical details^3^.

Our study has some limitation. This study is partly retrospective (observation period from 2016 to 2018) and partly prospective (observation period from 2019 to 2021), so we cannot fully conduct an effective analysis between anatomical variations of vascular anatomy with postoperative complications in a group of cases that were retrospectively analyzed.

## CONCLUSION

Personalized preoperative analysis of 3D-CT angiography is a key pattern in assessment of vascular anatomy and can potentially show the complexity of future lymphadenectomy, reduce intraoperative time for identifying key landmarks, and develop an individualized surgical strategy. Personalized 3D-CT assessment can potentially significantly reduce the risk of AL. To solve this problem, new studies and further standardization are needed.
